# Case report: Rare among ultrarare—Clinical odyssey of a new patient with Ogden syndrome

**DOI:** 10.3389/fgene.2022.979377

**Published:** 2022-09-05

**Authors:** Jagoda Hofman, Michal Hutny, Karolina Chwialkowska, Urszula Korotko, Karolina Loranc, Anna Kruk, Urszula Lechowicz, Adriana Rozy, Pawel Gajdanowicz, Miroslaw Kwasniewski, Malgorzata Krajewska-Walasek, Justyna Paprocka, Aleksandra Jezela-Stanek

**Affiliations:** ^1^ Students’ Scientific Society, Department of Paediatric Neurology, Faculty of Medical Sciences in Katowice, Medical University of Silesia, Katowice, Poland; ^2^ IMAGENE.ME SA, Bialystok, Poland; ^3^ Centre for Bioinformatics and Data Analysis, Medical University of Bialystok, Bialystok, Poland; ^4^ Department of Genetics and Clinical Immunology, Institute of Tuberculosis and Lung Diseases, Warsaw, Poland; ^5^ Department of Clinical Immunology, Wroclaw Medical University, Wroclaw, Poland; ^6^ Department of Paediatric Neurology, Faculty of Medical Sciences in Katowice, Medical University of Silesia, Katowice, Poland

**Keywords:** Ogden syndrome, NAA10 gene, progeroid appearance, feeding difficulties, pulmonary infections

## Abstract

**Introduction:** The definition of ultra-rare disease in terms of its prevalence varies between the sources, usually amounting to ca. 1 in 1.000.000 births. Nonetheless, there are even less frequent disorders, such as Ogden syndrome, which up to this day was diagnosed in less than 10 patients worldwide. They present typically with a variety of developmental defects, including postnatal growth retardation, psychomotor delay and hypotonia. This disorder is caused by the heterozygous mutations in *NAA10* gene, which encodes N-alpha-acetyltransferase 10, involved in protein biosynthesis. Therefore, Ogden syndrome belongs to the broader group of genetic disorders, collectively described as *NAA10*-related syndrome.

**Case report:** We present a case of a Polish male infant, born in 39. GW with c-section due to the pathological cardiotocography signal. Hypotrophy (2400 g) and facial dysmorphism were noted in the physical examination. From the first minute, the child required mechanical ventilation - a nasal continuous positive airway pressure. For the first 27 days, the patient was treated in a neonatal intensive care unit, where a series of examinations were conducted. On their basis, the presence of the following defects was determined: muscular ventricular septal defects, patent foramen ovale, pectus excavatum, clubfoot and axial hypotonia. Child was then consequently referred to the genetic clinic for counselling. Results of the tests allowed the diagnosis of Ogden syndrome. In the following months the patient’s condition worsened due to the numerous pulmonary infections. Despite the advanced treatment including the variety of medications, the patient eventually died at the age of 10 months.

**Conclusion:** This case report presents a tenth patient diagnosed with Ogden syndrome reported worldwide. It expands the morphologic and clinical phenotype, emphasizing the possible severity of pneumonological disorders in these patients, which may pose a greater threat to a child’s life than more frequently described cardiovascular dysfunctions associated with this syndrome.

## 1 Introduction

One of the well conserved co-translational protein modifications is N-alpha-acetylation, influencing the proteins’ stability. The enzymes responsible for it are N-alpha-acetyltransferases (NATs) complexes (van Damme et al., 2011). Six NATs were found in humans, called respectively NatA-F. Their mechanism is transferring the acetyl group from acetyl-CoA to the N-end of target molecule, which in case of the NatA are small sidechains: Ser, Ala, Thr, Gly, Val, Cys (Dörfel and Lyon, 2015). This enzyme consists of a catalytic subunit: N-alpha-acetyltransferase 10 (Naa10), encoded by the *NAA10* gene (Xq28, MIM 300013), and an auxiliary anchoring-subunit: Naa15, encoded by *NAA15* gene (4q31.1, MIM 608000) (McTiernan et al., 2020).

Depending on the type and inheritance pattern of the mutation found in the individual, the patients with *NAA10* variants present with a wide spectrum of symptoms, described as *NAA10*-related syndrome (Wu and Lyon, 2018). Though its prevalence is low, there is a growing number of patients reported with its subtypes, which expand its clinical variability (Cheng et al., 2019). Its phenotype ranges from mild intellectual impairment to the lethal combination of growth failure, delayed psychomotor development and progeroid-like dysmorphic features, commonly known as Ogden Syndrome (OGDNS, Xq28, MIM 300855). The latter belongs to the ultra-rare diseases, as up to date less than 10 patients have been diagnosed with this disorder worldwide. The mutation causing this syndrome is a heterozygous X-linked recessive missense substitution, leading to change from serine to proline in a protein sequence - p(Ser37Pro) (Popp et al., 2015; Wu and Lyon, 2018). In *NAA10*-related syndrome various other mutations were found, some of them presenting an X-linked dominant inheritance model (Casey et al., 2015). This case report presents a tenth reported patient diagnosed with OGDNS worldwide, and to our best knowledge the first one described in the Polish population. Contrary to previous patients with OGDNS, the boy in a current study showed a less severe phenotype of cardiovascular defects, in turn experiencing respiratory distress episodes and recurrent upper and lower respiratory tract infections, which eventually were responsible for his death.

## 2 Case report

### 2.1 Patient information

A male infant of healthy non-consanguineous Caucasian parents (31-year-old mother, 33 years-old father) was born as a second child (caesarean section) due to the pathological cardiotocography (CTG) trace, after 39 weeks of pregnancy complicated with maternal hypothyroidism and gestational diabetes. During the labour occurred a leakage of greenish amniotic fluid. The Apgar’s score was measured as 7/8/8/8—patient required continuous positive airway pressure ventilation (CPAP), which after 2 h led to stable respiration. The boy was hypotrophic, weighing 2400 g. The remaining birth parameters were as follows: length—48 cm; head circumference—32 cm; chest circumference—31 cm.

Dysmorphic features were found in the physical examination, including ocular hypotelorism, gothic palate, retrognathism, low-set ears and hairline, hypertrichosis, broad and flat nasal bridge, wrinkled facial skin, pectus excavatum. Developmental defects were also present in the extremities, including the arachnodactyly, clubfoot. Due to these features and the initial respiratory instability, the patient was hospitalised for 27 days at the Neonatal Intensive Care Unit, Institute of Mother and Child, Warsaw, Poland. There he was consulted neurologically, orthopaedically and ophthalmologically, which allowed the diagnosis of axial hypotonia and limbal retinal immaturity.

In the course of genetic examination a hemizygous variant on chromosome X in *NAA10* gene - p(Ser37Pro) was identified. This variant is present in three other independent families, in which the children with OGDNS clinical phenotype were previously found (Rope et al., 2011; Gogoll et al., 2021). In presented patient it was inherited from the mother, who does not present any relevant clinical findings. Similarly patient’s sister does not present any symptoms characteristic of OGDNS. A written informed consent for the genetic examination as well as further publication of the case description in scientific journal has been signed by the proband’s parents.

### 2.2 Clinical findings

#### 2.2.1 Respiratory infections

The most prominent clinical issue in the presented case was recurring infections of the upper and lower respiratory tract. In week 6. the patient was admitted to the hospital due to the upper airway infection after 2 days of enteroviral rhinitis. An additional clinical finding, which proved to be independent of the pneumonological symptoms was subtle intercostal retractions.

In the 12 week the patient presented with fever and cough and was again referred to the hospital with suspected pneumonia. The left-sided crackles were present in auscultation. Biochemical examination showed increased levels of inflammatory markers and microcytic anaemia. After 3 days of hospitalisation, the patient developed tachypnoea, severe dyspnoea, wheezing in the lung fields, and extended inspiration phase.

#### 2.2.2 Cardiovascular system

Initially, apart from a minor right-sided ventricular hypertrophy, ventricular septum defect (VSD), resulting in a mild left-right shunt, and haemodynamically irrelevant tricuspid valve insufficiency, the boy presented a physiological cardiac morphology and mechanic parameters. During his hospitalisation at the age of 4 weeks, the elevated levels of cardiac insufficiency markers (NT-proBNP: 10,569.0 pg/ml; Troponin T: 72.2 ng/L) were observed together with an enlarged cardiac silhouette in chest x-ray (CXR) imaging. Electrocardiographic findings of a single examination conducted at the age of 14 weeks in the course of cardiological consultation included partial right bundle branch block and low voltage of QRS complexes in limb leads. Despite the above observations, no signs of cardiac dysfunction were found in echocardiography (ECG). In accordance with previously observed left-right atrial shunt, the presence of patent foramen ovale (PFO) was discovered.

#### 2.2.3 Lipid metabolism

The first-day measurements of cholestasis markers raised suspicion, as the γ-glutamyltransferase (GGT) values significantly exceeded the reference range (1264 U/L), though in the following days these values gradually lowered. On the 5. day the boy presented with symptoms of cholestatic jaundice and the elevation of direct bilirubin to 2.2 mg%. The remaining hepatic biomarkers remained within reference range, whereas bilirubin and GGT values lowered to respectively 1.26 mg% and 217 U/L, which correlated with the improvement of the clinical symptoms. Ultrasound imaging did not detect any abnormalities in the size of the abdominal organs, and no signs of inflammation or other pathological processes were found.

**FIGURE 1:**
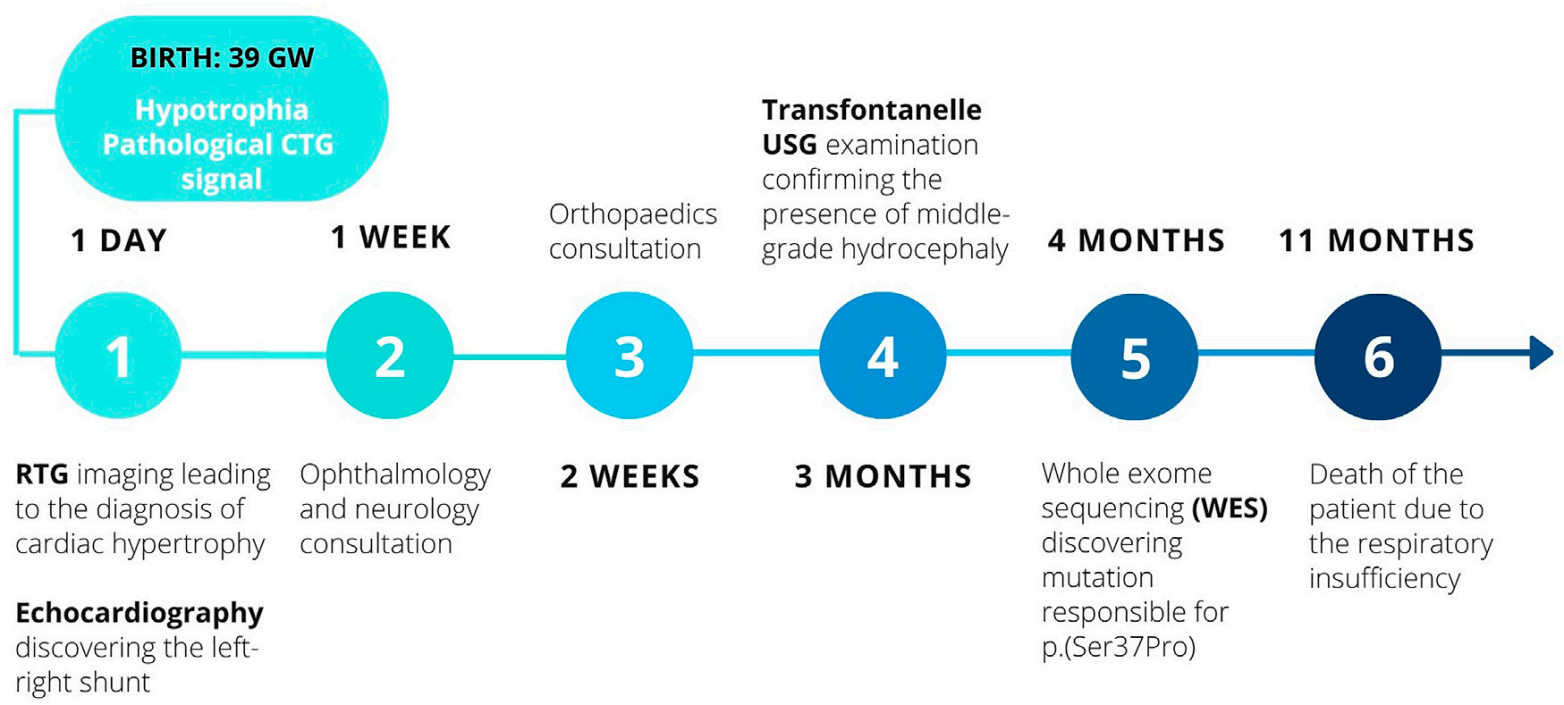
The timeline of diagnostic and therapeutic interventions.

In order to summarise the chronology of the diagnostic process and the therapeutic interventions, the timeline of patient’s history is presented below in Figure 1.

### 2.3 Diagnostic assessment

In the cases of single-gene diseases e.g. OGDNS the genetic examination has the most prominent role in diagnosis, as it allows the discovery of the exact causative mutation. Nevertheless, the classical clinical diagnostic measures are crucial for monitoring the patient’s condition and for controlling the eventual development of complications.

#### 2.3.1 Genetic studies

Samples for genetic analysis using whole exome sequencing (WES) have been collected from proband and his biological parents. Buccal swabs were collected using SK-3S Isohelix SWAB KIT (Cell Projects Ltd, United Kingdom) and preserved in Isohelix BuccalFix (Cell Projects Ltd, United Kingdom). DNA was isolated with the XME-50 Xtreme DNA kit for isolating purified DNA (Cell Projects Ltd, United Kingdom). The quantity and purity of gDNA was evaluated using spectrophotometer NanoPhotometer® N50 (Implen, CA, United States) and Qubit 2.0 Flurometer (Thermo Fisher Scientific, MA, United States). Exome libraries were prepared using the commercial Illumina Nextera DNA Flex for Enrichment reagent kit accordingly to the manufacturer’s instructions (Illumina, CA, United States). The Illumina CEX v1.2 panel (Illumina, CA, United States) was used for hybridisation, with the added custom panel probes covering significant non-coding regions as well as mitochondrial genome. Patient libraries were tagged with unique dual index barcodes during library preparation. The quality and insert sizes of the prepared WES libraries was assessed using the TapeStation device (Agilent Technologies, CA, United States) and the High Sensitivity DNA ScreenTape kit (Agilent Technologies, CA, United States). The concentration of fragments in the libraries was measured with Qubit 2.0 Flurometer (Thermo Fisher Scientific, MA, United States). The prepared WES libraries were pooled and diluted accordingly to manufacturer’s protocol sequenced with the Illumina NovaSeq 6000 system (Illumina, CA, United States) using 2 × 100 bp (200bp) paired-end (PE) reads.

**FIGURE 2:**
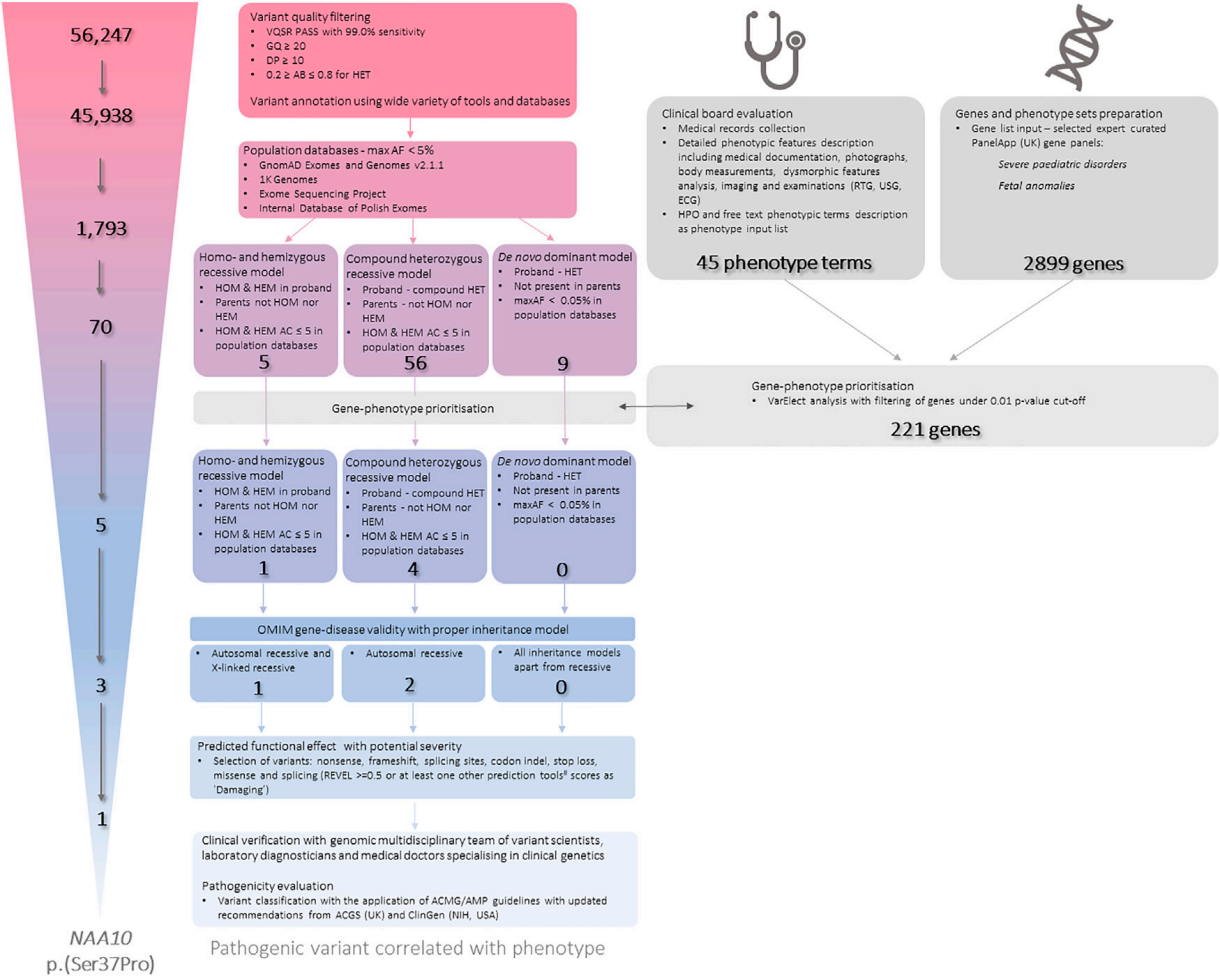
The workflow of the Whole Exome Sequencing (WES) data processing, filtration, annotation and final interpretation.AB—Allele Balance; AF—Allele Frequency; C—Allele Count; DP—Depth of Coverage; GQ—Genotype Quality; HEM—Hemizygous; HET—Heterozygous; HOM—Homozygous; HPO—Human Phenotype Ontology; max—maximum; REVEL—Rare Exome Variant Ensemble Learner; VQSR—Variant Quality Score Recalibration. ^#^—predictive tools that were used: CADD; BayesDel noAF; FATHMM-MKL; MutationTaster; Polyphen2; SIFT.

Whole exome sequencing (WES) data was analysed according to the Genome Analysis Toolkit (GATK) recommendations (DePristo et al., 2011). Reads were mapped to a reference genome (GRCh38) using Burrows-Wheeler aligner (BWA) v0.7.17 (Li and Durbin, 2010). Duplicates were removed using Picard v2.10.9. Variant calling was carried out using GATK v4.1.4.0 HaplotypeCaller in GVCF mode. All samples were joint-genotyped using GATK GenotypeGVCFs and subjected to filtering using GATK Variant Quality Score Recalibration (VQSR). The samples were subjected to additional filtering, during which low quality variants were removed, as shown below in Figure 2. Low quality variants were defined as variants with genotype quality of less than 20, a depth of less than 10, or an allele balance of less than 0.2 or more than 0.8 if heterozygous. The samples were subjected to quality control in several stages of analysis. The quality of raw reads was assessed by FastQC v0.11.5 (Wingett and Andrews, 2018) and MultiQC v1.7 (Ewels et al., 2016). Qualimap v2.2.1 (Okonechnikov et al., 2016) and Samtools v1.9 (Danecek et al., 2021) were used to assess the quality of the mapping to the reference genome. The quality of the identified genotypes was controlled by in-house scripts. Average depth of coverage was 100.94× (proband), 133.08× (mother), 111.59× (father). VCF files were annotated using various clinical and genomic databases and annotation tools. Analysis was performed in trio setting with proband and his biological parents. Data has been filtered according to minor allele frequencies, variant effects and known annotations in databases and journal publications in different settings accordingly to expected inheritance and variant parameters (Figure 2.). Variants were also subjected to gene prioritisation based on gene-phenotype correlation. An extensive phenotypic data were collected and evaluated including medical records collection and detailed phenotypic features description including medical documentation, photographs, body measurements, dysmorphic features analysis, imaging and examinations (RTG, USG, ECG). Proband’s phenotype has been described in 45 phenotypic terms, mainly in accordance to the HPO (Human Phonotype Ontology) nomenclature. VarElect (Stelzer et al., 2016) has been used to prioritise genes from two PanelApp (United Kingdom) gene sets: Severe pediatric disorders and Fetal anomalies. Genes significant under a cut-off of *p*-value≤0.01 were further selected and used as an additional filter. Identified variants for were also manually reviewed in the Integrative Genomic Viewer (IGV) (Thorvaldsdóttir et al., 2013). Detected variants were selected for further expert curation. In the whole process of variant filtration, curation and interpretation, a multidisciplinary expert team has been involved, consisting of: variant scientists, laboratory diagnosticians, and medical doctors specialising in clinical genetics.

We have identified a hemizygous variant on chromosome X in *NAA10* gene - NM_003,491.4: c.109 T>C p(Ser37Pro) [rs387906701; GRCh38 chrX:153934388:G:C]. The variant p(Ser37Pro) has been inherited from the unaffected mother. It has been covered 87× in the proband’s sample (AB = 100%) and 155× in his mother’s sample (AB = 35%). We have evaluated and interpreted the variant accordingly to the internal standard operation procedures based on the ACMG/AMP (American College of Medical Genetics/Association for Molecular Pathology, United States) (Richards et al., 2015; Jarvik and Browning, 2016) guidelines with ClinGen (The Clinical Genome, NIH, United States) (Rehm et al., 2015; Rivera-Muñoz et al., 2018; Brnich et al., 2019) and ACGS (The Association for Clinical Genomic Science, United Kingdom) recommendations. The variant was interpreted as pathogenic. *In vitro* functional assays provide an evidence for reduced protein activity and substrate binding resulting in acetylation inhibition (pathogenic strong 3 (PS3); PS3_Supporting) (Rope et al., 2011; Myklebust et al., 2014). Variant is located in a mutational hot spot and well-established functional domain (acetyltransferase GNAT domain; pathogenic moderate 1 (PM1)), as well as missense constrained gene region (pathogenic supporting 2 (PP2)). The variant is completely absent in population databases: GnomAD (v2.1.1; v3.1.2), ExAC and BRAVO, as well as internal database of Polish exomes (>3000 samples) (PM2). Multiple computational tools predicted a deleterious effect of the variant (PP3; CADD score: 32; BayesDel noAF: 0.2385—damaging; FATHMM-MKL: 0,9039 - damaging; MutationTaster: 1 - disease causing; Polyphen2: damaging; SIFT: 0,001—damaging; while REVEL score was not shifted towards pathogenicity or benign prediction with the value of 0.5569). Three other independent families with OGDNS clinical phenotype have been identified with this variant (PS4_Moderate) (Rope et al., 2011; Gogoll et al., 2021). The variant segregated with the phenotype in multiple individuals in two families (PP1_Strong) (Rope et al., 2011). Proband exhibits highly gene-specific phenotype (PP4_Strong). The variant has been deposited previously in clinical genomic databases: ClinVar (one pathogenic submission; Variation ID: 29,927); LOVD (pathogenic); HGMD (DM; acc. no. CM115549).

#### 2.3.2 Imaging

The discovery of alarming signs in the RTG imaging, which suggested the presence of cardiac hypertrophy, indicated the necessity of conducting an ECG examination. The limitation of this investigation were the narrow intercostal spaces and the thoracic malformation. The test ruled out the hypertrophy, but confirmed the PFO (initially described as atrial septal defect (ASD)), VSD and the left-right shunt. It also suggested the possibility of coronary arterial fistulas (CAF), which could not be confirmed due to death of patient.

Ultrasonography (USG) was applied for examining other regions—abdominal, single pelvic and femoral head USGs did not discover any abnormalities concerning the morphology of organs located in these regions. Imaging of scrotum ruled out cryptorchidism, but confirmed the presence of left-sided epidydimal cyst (12 × 5 mm). Due to the physiological size of anterior and posterior fontanelles it was possible to conduct a transfontanellar USG, which together with consequent magnetic resonance imaging (MRI) discovered the evolution of the ventricular system, from the physiological size to the middle-grade hydrocephalus in August.

### 2.4 Therapeutic intervention

#### 2.4.1 Nutrition and feeding

During his short lifetime the patient developed a number of metabolic and nutritional problems, which had to be addressed. From the first day of life the boy presented with icterus, hepatic cholestasis and elevated levels of cholestatic marker (GGT). Therefore, ursodeoxycholic acid (UDCA) was included in the patient’s treatment scheme, as well as the supplementation of fat-soluble vitamins—A, E and D (calcifediol). Additionally, due to anaemia of prematurity, the patient was prescribed oral iron supplementation and vitamin K, which was continued throughout the whole patient’s life.

Due to the structural alterations in the temporomandibular joint (TMJ) region, resulting in the incorrect mandibular mobility, as well as the tension alterations in the musculature of the oral cavity, the patient was fed parenterally during the first 3 days, combined with enteral nutrition since the 2nd day of the first hospitalisation. The patient was referred to ergotherapy. Though the horizontal mandibular mobility was not achieved, the patient learned to efficiently use the dummy.

#### 2.4.2 Posture correction

After the orthopaedic consultation, the patient was treated with feet taping, extension and positioning exercises. Kinesiotaping was not applied due to the lack of possibility of improving the defect with this technique. The therapy was aimed at the redistribution of tensions, and improvement of dynamic stabilisation of soft tissues, including the plantar aponeurosis as well as the tendons and periarticular structures in the chosen feet sections. The physiotherapy was also applied in thoracic region, which showed severe malformations. Muscles of the pectoral girdle, and the pectoral, scapular and glenohumeral musculature were corrected in terms of tension distribution and movement range.

#### 2.4.3 Infections

In the first 2 days after birth the patient was given an empiric antibiotic treatment, consisting of ampicillin and gentamicin. Due to the gastrointestinal *Candida albicans* infection, the boy received four daily p.o. nystatin doses in span of a week. During the second hospitalisation the upper respiratory tract infection was treated symptomatically with 0.9% NaCl nebulisation, followed by drainage of nasal cavity. In case of third hospitalisation, when the patient suffered from severe pneumonia, he was treated with a range of medicaments: fenoterol + ipratropium bromide, antibiotics, budesonide, prednisolone.

No contraindications for vaccinating were found, therefore the patient was immunized with dedicated vaccines in 1. and 4. week of life. No adverse reactions were observed.

### 2.5 Follow-up and outcomes

Although in the hospital setting the pulmonary infections were eventually controlled and the patient could be discharged, the patient’s condition worsened, leading to his death at the age of 10 months. The direct cause was respiratory failure in the course of respiratory tract infection.

## 3 Discussion

The patient presented in this study shared some of the main features of other children diagnosed with OGDNS, though to some extent. Despite the very low quantity of OGDNS patients worldwide, all of these children presented the distinctive details of their phenotype, both in terms of their morphology, as well as the clinical image (Rope et al., 2011; Gogoll et al., 2021). One of the key symptoms of this syndrome, apart from the typical facial appearance, are hypotonia and feeding difficulties, often resulting from dysphagia. A significant part of this population had cholestasis-associated features, including the elevated cholestatic biomarkers levels and icterus, both of which were found in the current patent.

The analysis of malformations present in the respective ten cases of OGDNS is summarized below in Table 1.

**TABLE 1:** The summary of the most frequent dysmorphic features of patients with OGDNS.

Study	Family	Patient	Large fontanelles	Progeroid facial appearance	Prominent eyes	Large ears	Flared nares	Narrow palate	Prominent nose
*Current study*	1	I-1	−	+	−	−	−	−	+
Gogoll et al., (2021)	1	I-1	−	+	+	+	+	+	+
Rope et al., (2011)	1	II-1	+	−	−	+	+	+	+
	1	II-6	+	+	+	+	+	−	−
	1	III-7	+	−	−	−	−	−	−
	1	III-4	+	−	+	+	+	−	−
	1	III-6	+	−	+	+	−	−	−
	2	II-1	−	+	+	+	−	−	−
	2	III-2	−	−	+	−	−	−	−
	2	III-4	−	−	−	−	−	−	−

Study	Family	Patient	Short neck	Hypertelorism	Micrognathia	Retrognathia	Metatarsus valgus	Pectus excavatum
*Current study*	1	I-1	−	−	−	+	+	+
Gogoll et al., (2021)	1	I-1	+	+	+	+	−	−
Rope et al., (2011)	1	II-1	+	−	−	−	+	−
	1	II-6	+	−	+	+	+	−
	1	III-7	−	+	+	−	−	−
	1	III-4	+	+	+	+	−	−
	1	III-6	−	+	+	+	−	−
	2	II-1	−	−	−	−	−	−
	2	III-2	−	−	−	−	−	−
	2	III-4	−	−	−	−	−	−

The features fitting the typical phenotype of OGDNS which were found in the present patient included characteristic progeroid facial appearance, hypertrichosis and low hairline. Features appearing less frequent in the population of these patients, but also present in the presented case were retrognathia, prominent nose and metatarsus valgus (here taking the form of clubfoot). A distinct malformation, which was not found in the previous reports, and is also significant for the pathogenesis of pulmonary symptoms characteristic for presented patient is pectus excavatum.

Although the patient’s cardiovascular history was not insignificant, the malformations (VSD, PFO, tricuspid valve insufficiency) and cardiac hypertrophy (right ventricle) were not haemodynamically relevant. In comparison to other children with OGDNS, the boy presented herein did not suffer from any cardiovascular complications, nor were they the causative factor for his death. It cannot be however ruled out, that if the patient lived long enough, his heart defects could lead to the relevant haemodynamic dysfunctions. The comparison of the most frequent cardiovascular features of all patients with OGDNS are presented below in Table 2.

**TABLE 2:** Cardiovascular defects and dysfunctions present in the children with OGDNS.

Study	(Family)	Patient	Cardiac hypertrophy	Cardiac insufficiency	ASD	VSD	PFO	PDA	Pulmonary stenosis	Arrhytmias
*Current study*	1	I-1	+	−	−	+	+	−	−	−
*Gogoll et al.*(Gogoll et al., 2021)	1	I-1	−	−	+	−	+	−	−	−
*Rope et al.*(Rope et al., 2011)	1	II-1	+	+	−	−	−	−	+	+
	1	II-6	−	−	−	−	−	+	−	−
	1	III-7	+	+	−	−	+	+	−	−
	1	III-4	+	+	+	+	+	−	+	+
	1	III-6	−	−	−	−	−	−	−	−
	2	II-1	−	−	−	−	−	−	−	−
	2	III-2	−	−	−	−	−	−	−	+
	2	III-4	−	−	−	−	−	−	−	+

ASD, atrial septal defect; PDA, patent ductus arteriosus; PFO, patent foramen ovale; VSD, ventricular septal defect.

The boy presented in this case is not the first one to suffer from frequent respiratory tract infections or respiratory distress, nevertheless, the pneumonological symptomatology is the less frequent component of the typical OGDNS phenotype. In none of the previous patients these disorders were equally severe, up to the point of leading to the patient’s death as in the presented boy.

In conclusion, this case report of the first described children with OGDNS found in the Polish population, as well as tenth child reported worldwide, widens the previously known range of phenotypical presentations of this syndrome. Cardiovascular defects and disorders, usually posing a major threat to patients’ health and often being the causative factor for their death, in the presented boy were mostly asymptomatic. Contrary, the presented case emphasizes the importance of respiratory disorders and their influence on a patient’s condition.

It also indicates that even among the patients with *NAA10*-related syndrome, who share the same pathophysiological mutation, the symptomatology range is not fully described, neither are the exact genotype-phenotype correlations adequately studied. The latter is an important issue for further research in the field of *NAA10*-related syndrome, as the answer concerning these associations would be crucial for further development of prenatal diagnostic and prognostic measures for these disorders. As evidenced by numerous cases of other *NAA10*-related syndrome subtypes, it affects not only male patients, but also females (McTiernan et al., 2018; Maini et al., 2021). All these patients may present with milder phenotype, such as non-syndromic intellectual disability (McTiernan et al., 2018), up to significant malformations (septal defects, microcephaly) (Ree et al., 2019; Afrin et al., 2020) and severe functional disorders—arrhythmias, developmental delays (Casey et al., 2015; Ree et al., 2019). In siblings of some of the affected children, who were carriers of the mutation, was found a milder phenotype, consisting of individual malformations (Afrin et al., 2020). Though in above cases the defects do not lead to early lethality, they leave a significant clinical mark on the quality of patients’ lives.

## Data Availability

The raw data was produced in the realm of patient’s diagnostic procedures. The data was collected and stored under GDPR rules. Raw genetic data may be shared upon request from the applicant after consent from the patient’s family.
